# Producing highly effective extracellular vesicles using IBAR and talin F3 domain fusion

**DOI:** 10.1080/19768354.2024.2353159

**Published:** 2024-05-18

**Authors:** Joonha Lee, MinHyeong Lee, Jiyoon Kim, Eun-Gyung Cho, Chungho Kim

**Affiliations:** a Department of Life Sciences, Korea University, Seoul, Republic of Korea; b Department of Molecular Genetics, University of Toronto, Toronto, Canada; c Consumer Health 2 Center, CHA Advanced Research Institute, Bundang CHA Medical Center, Seongnam, Republic of Korea

**Keywords:** Extracellular vesicle, integrin, talin, curvature, IBAR

## Abstract

Extracellular vesicles (EVs), transporting diverse cellular components, play a crucial role in intercellular communication in numerous physiological and pathological processes. EVs have also been recognized as a drug delivery platform for therapeutic purposes and cell-free regenerative medicine. While various approaches have focused on increasing EV production for efficient use therapeutic use of EVs, enhancing the quality of EVs, such as ensuring efficient uptake by their target cells, has not been widely explored. In this study, we linked a negative membrane curvature-forming inverse BAR (IBAR) domain with an integrin β tail-binding talin F3 domain to create the IBAR-F3 fusion protein. We observed that IBAR-F3 can trigger filopodia-like membrane protrusions and attract integrins to those protrusion-rich regions, when expressed in Chinese hamster ovary cells expressing integrin αIIbβ3. Surprisingly, the expression of IBAR-F3 also induced a robust production of EVs, which were then efficiently taken up by nearby cells in an integrin-dependent manner. Moreover, IBAR triggered integrin activation, presumably by inducing negative membrane curvature that likely disrupts the interaction between the integrin α and β transmembrane domain. Therefore, we suggest that IBAR-F3 should be utilized to promote both EV production and efficient uptake mediated by integrins. Furthermore, the negative curvature-inducing integrin activation suggests that integrins on EVs can be activated by the nanoscale change in the curvature of the EV without the need for conventional machinery to activate integrin inside the EVs.

## Introduction

Extracellular vesicles (EVs) are cell-derived membrane vesicles, which include exosomes originating from intraluminal vesicles and microvesicles shed from the plasma membrane (Colombo et al. 2014; van Niel et al. 2018). EVs carry various cellular components, including lipids, nucleic acids, and proteins, and upon uptake by their target cells, they can deliver these components, serving as an effective tool of intercellular communication in many physiological and pathological processes (Zaborowski et al. 2015; Kalluri and LeBleu 2020; Kita and Shimomura 2022; Kim et al. 2024). Due to their inherent *in vivo* delivery capabilities, numerous attempts have been made to utilize EVs as a gene, protein, or drug delivery platform for therapeutic purposes (Wiklander et al. 2019; Herrmann et al. 2021). Additionally, stem cell-derived EVs, presumably loaded with a range of restorative factors such as growth factors, are considered promising cell-free materials for regenerative medicine treatment (De Jong et al. 2014; Allan et al. 2020).

EVs’ quantity and quality are critical for their efficiency in therapeutic uses (Esmaeili et al. 2022). To improve the yield of EVs, various approaches have been utilized (Syromiatnikova et al. 2022), including maintaining donor cells under acidic conditions (Boussadia et al. 2018) or 3D conditions (Cha et al. 2018; Rocha et al. 2019), applying low-level electricity (Fukuta et al. 2020), and extruding cells through porous filters to collect EV-like vesicles (Thone and Kwon 2020). On the other hand, the quality of EVs, specifically their efficient uptake by recipient cells, appears to be dependent on cell adhesion molecules such as integrins (Lin et al. 2023). Indeed, many proteomics-based approaches have revealed that integrin family adhesion molecules and their ligands are found in EVs and involved in the EV uptake into target cells (Hoshino et al. 2015; Vajen et al. 2017; Whitham et al. 2018; Guo et al. 2019), highlighting integrins’ critical role in the cell uptake efficiency of EVs. Therefore, it is desirable to develop a method that makes it possible to ensure the inclusion of integrins in EVs and collect a large quantity of EVs.

Integrins’ affinity to their ligands is dynamically changed, depending on integrins’ conformation. During the ‘integrin activation’ process, which is regulated by intracellular signaling pathways, the bent extracellular domain of integrin is extended and the ligand affinity becomes high (Luo et al. 2007; Kim et al. 2011b). In the signaling pathway to activate integrin, talin, a cytoskeletal protein, binds to the cytoplasmic tail of the integrin β subunit (Lagarrigue et al. 2016). This interaction, in turn, alters the topology of the transmembrane domain of integrin β and disrupts the interaction between the transmembrane domains of α and β subunits, triggering integrin activation (Kim et al. 2009; Kim et al. 2011a; Kim et al. 2012; Kim and Kim 2013). This feature of integrin activation suggests that incorporating integrin activation machinery into EVs should facilitate integrin-mediated EV uptake.

Talin contains an N-terminal head domain (talin head domain, THD) subdivided into the F0, F1, F2, and F3 domains (Campbell 2010). Of these subdomains, the F3 domain directly binds to the integrin β tails and induces integrin activation (Calderwood et al. 2002; Bouaouina et al. 2008). Previously, we reported that fusion of the F3 domain to the epsin N-terminal homology (ENTH) domain greatly activates integrin αIIbβ3 (Kim et al. 2020). The ENTH domain at the membrane contact induces an inward positive membrane curvature, a characteristic observed in endocytosis (Ford et al. 2002), and the integrin activation effect of the fusion protein of ENTH and F3 domain is abolished, in case mutant ENTHs unable to induce the membrane curvature is used. Thus, it was proposed that the integrin activation caused by ENTH-F3 fusion results from the disruption of the integrin α–β transmembrane domain interaction induced by ENTH-mediated membrane curvature (Kim et al. 2020).

In this study, we investigated the impact of outward negative membrane curvature, exhibiting a curvature direction opposite to that induced by ENTH and commonly observed in membrane protrusion near integrin activation sites, on EV biogenesis. For this purpose, we adopted the inverse BAR (IBAR) domain inducing outward negative membrane curvature (Nepal et al. 2021). Surprisingly, transfection of the IBAR domain alone induced a robust generation of EVs. Moreover, the IBAR-F3 fusion protein led not only to integrin activation but also to an efficient uptake of the EVs into recipient cells. Therefore, we propose that the IBAR-F3 fusion protein increase the EV production through membrane curvature change and the uptake efficiency by incorporating ‘active integrins’.

## Materials and methods

### Plasmids

The IBAR domain was obtained through PCR amplification from cDNA encoding human insulin receptor substrate protein 53 (IRSp53), also known as BAIAP2, purchased from the Korea Human Gene Bank at the Medical Genomics Research Center, KRIBB, Korea. The PCR-amplified IBAR domain was then integrated into pcDNA3.1/green fluorescence protein (GFP) or F3-GFP constructs (Kim et al. 2020) with a GGGGS linker at the C-terminal end of the IBAR domain, producing IBAR-GFP or IBAR-F3-GFP constructs. IBAR(K142E/R145E) or F3(K322D) mutation was generated by PCR-mediated site-directed mutagenesis. PCR-amplified THD and its F3 domain-deletion mutant, THD(ΔF3), were ligated into the pcDNA3.1/N-Ub construct to generate N-Ub-THD and N-Ub-THD(ΔF3). The N-Ub-THD(K322D) mutant was generated by PCR-mediated site-directed mutagenesis.

### Cell lines

Chinese hamster ovary cells stably expressing integrin αIIbβ3, CHO/αIIbβ3 cells, were established as described previously (Kim et al. 2016; Kim et al. 2020). Cells were cultured in Dulbecco’s modified Eagle’s medium (DMEM, Thermo Fisher) supplemented with 10% (v/v) fetal bovine serum (FBS), 2 mM L-glutamine, non-essential amino acids, and penicillin – streptomycin and maintained in an incubator at 37°C with 5% CO_2_. For the mammalian two-hybrid assay, CHO cells were stably transduced by lentiviral infection using 5xGAL4UAS-GFP in pHR (Addgene) (Petschnigg et al. 2014) and the integrin β3 TMD-tail fused to the C-terminal half of ubiquitin (C-Ub) in pTet-On lentiviral vectors. The resulting CHO cells were used for the transient transfection of THD or its mutants fused to the N-terminal half of ubiquitin (N-Ub) constructs.

### Flow cytometry

CHO/αIIbβ3 cells were transfected with various constructs using PolyJet^TM^ In Vitro DNA Transfection Reagent (SignaGen Laboratories) following the manufacturer’s recommendations. When needed, eptifibatide (Santa Cruz Biotechnology) was added to a final concentration of 20 μM after transfection. One day after transfection, the cells were detached with trypsin-EDTA, spin-washed twice with DMEM, and assessed for GFP expression via flow cytometry using either FACS Calibur or FACS Symphony A1 (BD Biosciences). Alternatively, to measure PAC1 binding, washed cells were incubated with 4 μg/mL of PAC1 (BD Biosciences) for 30 min at room temperature with agitation. Following two washes, the cells were subsequently incubated with allophycocyanin-conjugated anti-mouse IgM antibody (Thermo Fisher Scientific) for 30 min at 4°C, washed again, and analyzed by flow cytometry.

### EV preparation

CHO/αIIbβ3 cells were transfected with IBAR DNA constructs. Three hours after transfection, the culture medium was replaced with low-serum media (DMEM containing 2% FBS). One day after transfection, the medium was collected and centrifuged at 1,000 g for 15 min at 4°C to eliminate cell debris. Then, the clarified supernatant was added with PEG-8000/NaCl solution (4% and 3% [w/v], respectively) and incubated overnight at 4°C with agitation. The resulting mixture was then centrifuged at 25,000 g for 20 min at 4°C, and the pellet was resuspended with phosphate-buffered saline (PBS) for use in subsequent experiments.

### Immunocytochemistry

CHO/αIIbβ3 cells were plated onto cover glasses and transfected with various DNA constructs. One day after transfection, the cells were fixed using a 3.7% formaldehyde solution in PBS, permeabilized with PBS containing 0.1% Triton X-100, and subsequently blocked using PBS supplemented with 5% goat serum, 0.03% gelatin, and 0.1% Triton X-100 for 30 min. Cells were then stained with anti-integrin αIIbβ3 (D57) followed by anti-mouse IgG-TRITC (Jackson Laboratory), each for 30 min. Rhodamine phalloidin (Thermo Fisher) was employed for actin staining when needed. The cover glasses with the stained cells were mounted onto slide glasses using Fluorescence Mounting Medium (Dako) containing Hoechst 33342 (Invitrogen) for nucleus visualization. Analysis was conducted using a fluorescence microscope (Ti-E, Nikon) equipped with a 100× (1.4 NA) plan-apochromat objective lens. Fluorescence images were captured using a charge-coupled device camera (DS-Qi2, Nikon) and deconvoluted using NIS-Elements AR software (Nikon).

## Results

### IBAR domains expressed in cultured cells can move to neighboring cells

**Figure 1.**
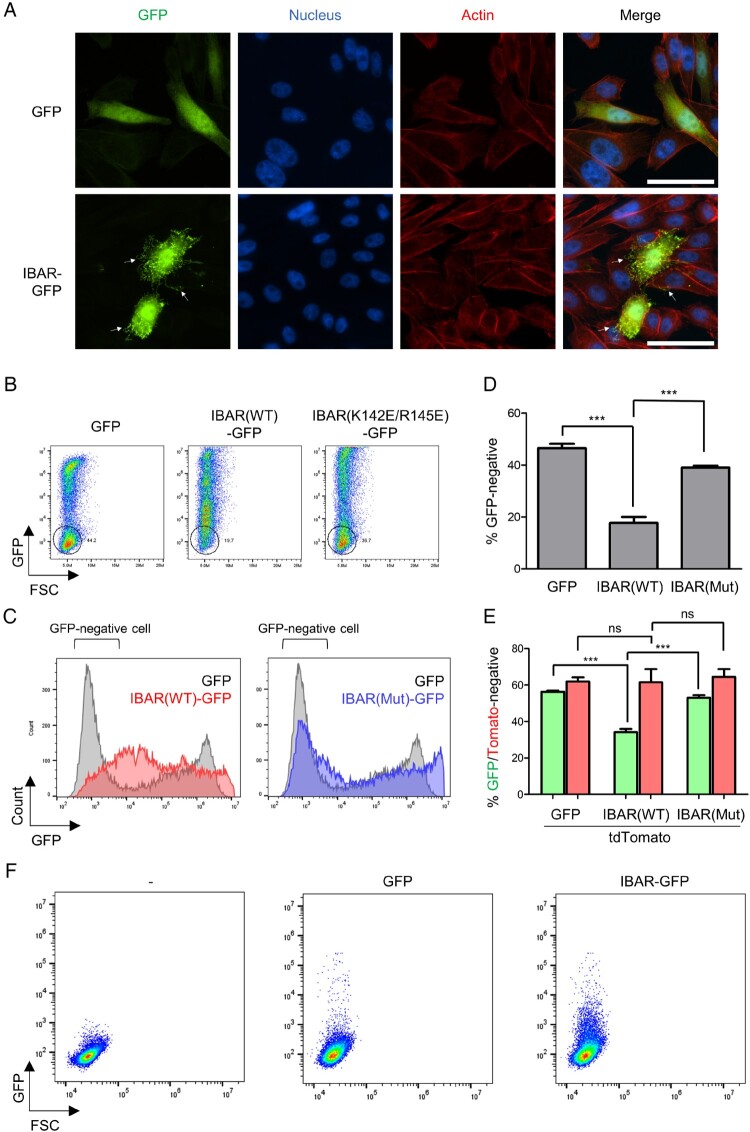
**Intercellular movement of the IBAR domain.** (A) CHO/αIIbβ3 cells transiently transfected with GFP or IBAR-GFP were stained with Hoechst 33342 and rhodamine phalloidin. Representative images are shown with filopodia-like membrane protrusions indicated with arrows. Scale bar, 50 µm. (B) CHO/αIIbβ3 cells transfected with GFP, IBAR wild type (WT)-GFP, or IBAR-GFP mutant were analyzed using flow cytometry. Representative dot plots showing GFP fluorescence and forward scatter from each sample are shown. The GFP-negative population is marked by black circles. (C) Histograms comparing GFP levels between IBAR-GFP or IBAR-GFP mutant (Mut)-transfected cells and GFP-transfected cells are shown. (D) The percentage of the GFP-negative population among the total cells is displayed as a bar graph (*n* = 3). Error bars indicate SD. ***p < 0.001 (Student’s t-test). (E) CHO/αIIbβ3 cells were transiently transfected with tdTomato and GFP-based constructs, and the percentages of the GFP-negative population, as in (D), and the tdTomato-negative population are displayed as bar graphs (*n* = 5). (F) Clarified cell culture medium from none-, GFP-, or IBAR-GFP-transfected CHO/αIIbβ3 cells was treated to fresh CHO/αIIbβ3 cells overnight, and the treated cells were analyzed using flow cytometry for their GFP expression. Representative dot plots are shown, as in (B). FSC, forward scatter; GFP, green fluorescence protein.

Membrane curvature is said to be negative when the second derivative of the membrane curvature is negative, leading to a decreasing first derivative. This negative curvature is notably observed in filopodia and vesicle budding (Jarsch et al. 2016; Ahn and Sun 2023). To induce negative curvature of the plasma membrane of cultured cells, we utilized the IBAR domain from IRSp53, a cytoskeletal protein that couples membranes with the cytoskeleton in actin-filled protrusions (Ahmed et al. 2010). We first fused the GFP into the IBAR domain to generate IBAR-GFP, and the transfected CHO cells stably expressing integrin αIIbβ3, CHO/αIIbβ3 cells, with the IBAR-GFP construct to test whether IBAR-GFP could induce a negative membrane curvature. Indeed, IBAR-GFP-transfected CHO/αIIbβ3 cells formed many filopodia-like membrane protrusions (Figure 1A), indicating that IBAR-GFP retains induction activity of negative curvature in CHO/αIIbβ3 cells. We performed flow cytometry of the IBAR-GFP transfected cells with a control of GFP-expressing cells. In GFP control, a population of untransfected cells appeared as low-fluorescence cells in dot plots (Figure 1B, left panel) or in histograms (Figure 1C, gray peak). Interestingly, however, in the flow cytometry of IBAR-GFP, the group of untransfected cells with low fluorescence mostly disappeared and the number of cells expressing high fluorescence noticeably increased. The shift from low fluorescence to high fluorescence was dependent on the curvature-forming ability of IBAR, because it was largely abolished by a curvature-defective mutation (K142E/R145E) (Suetsugu et al. 2006) in the IBAR domain (Figure 1B, right panel; Figure 1C, blue peak). To quantify these phenomena, we calculated the percentage of GFP-negative cells, within the region corresponding to the distinct round GFP-negative population observed in the dot plot of the GFP transfected condition (Figure 1C, black circle), in each experiment (Figure 1D). The percentage measured in the GFP transfection condition was more than 40%, which was reduced to about 20% by IBAR-GFP transfection and reversed again to the level of 40% by the curvature-defective mutation (Figure 1D). These results suggest that IBAR-GFP proteins expressed in transfected cells should be transferred to untransfected cells, and this transfer should be dependent on an intact IBAR domain having the negative curvature-forming ability. To further investigate this possibility, we co-transfected cells with IBAR-GFP and tdTomato. We found that the low fluorescence population was reduced in GFP signal, but not in tdTomato (Figure 1E). This result strongly indicates that IBAR domains expressed in cultured cells are actively transferred to neighboring cells, beyond simple transfer of intercellular materials. To confirm the IBAR-GFP transfer between cells in culture through culture medium, we cultured untransfected cells with the culture medium in which IBAR-GFP-expressing cells were incubated. We detected GFP fluorescence in some untransfected cells (Figure 1F, right). This clearly indicates that IBAR-GFP protein – or its cDNA-containing materials are released from transfected cells, and then taken up by recipient cells. In contrast, GFP fluorescence was minimally observed in cells treated with medium from non-GFP-transfected cells or GFP-transfected cells (Figure 1F, left and middle), excluding the possibility of the contamination of GFP or IBAR-GFP expressing cells in the medium.

### The IBAR domain enhances EV production

**Figure 2.**
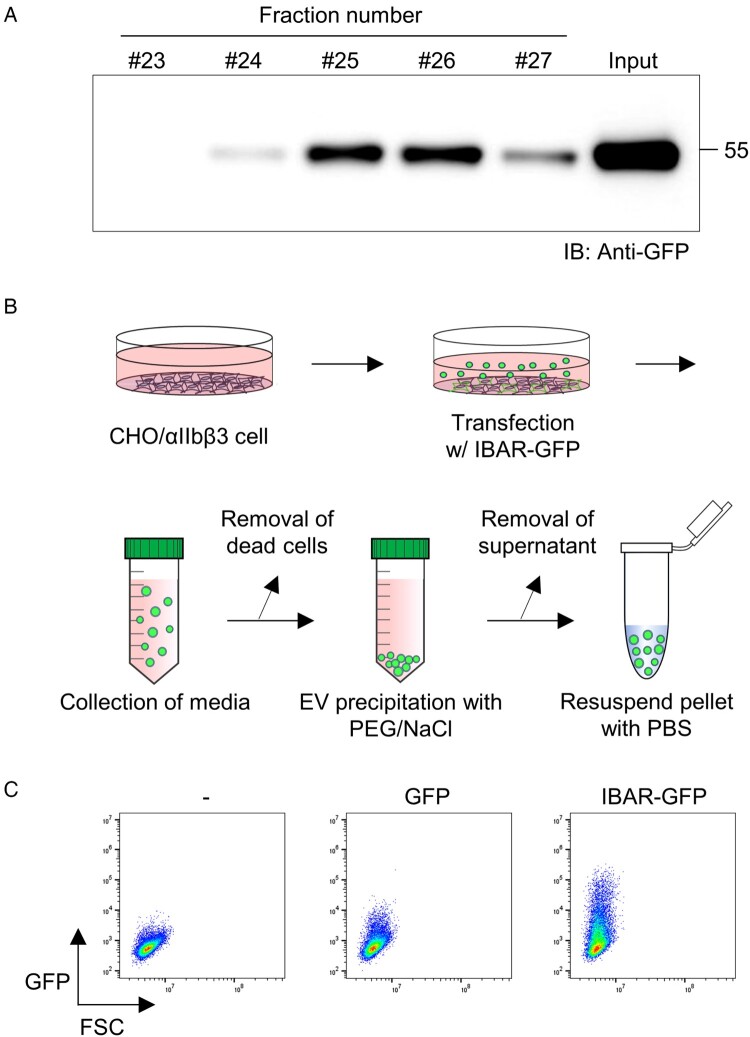
**EV production by the IBAR domain**. (A) The clarified conditioned media from IBAR-transfected CHO cells underwent fractionation using a size-exclusion column. Western blot analysis employing an anti-GFP antibody was conducted on both the flow-through fractions and the conditioned media prior to fractionation (input). The conditioned media before fractionation was loaded as input. IB, immunoblot. (B) A schematic illustration of the EV preparation process from IBAR-transfected cells is shown. (C) Conditioned media from GFP- or IBAR-GFP-transfected CHO/αIIbβ3 cells were collected, and EVs were purified from the media, as in (B). Purified EVs were added to fresh CHO/αIIbβ3 cells and incubated overnight. The EV-treated cells were analyzed by flow cytometry, and dot plots showing GFP fluorescence and forward scatter are shown. FSC, forward scatter; GFP, green fluorescence protein.

The observation that the IBAR domain can induce negative membrane curvature (Figure 1A) and can be transferred to neighboring cells led us to hypothesize that IBAR may generate membrane protrusions cleaved off to form EVs, which can then transfer IBAR-GFP to untransfected neighboring cells. Supporting this hypothesis, a recent study suggested that another IBAR domain from MIM, a cytoskeletal protein known for inducing cellular protrusions, facilitates EV formation by mediating the scission of filopodia (Nishimura et al. 2021). To test the hypothesis, we first applied size-exclusion chromatography to the cell culture media of the cells transfected with IBAR-GFP to fractionate EVs carrying IBAR-GFP. We detected green fluorescence in fractions corresponding to sizes larger than 440 kDa (Figure 2A), indicating that IBAR-GFP is present in a large complex in the media. Subsequently, we purified EVs from the media of MOCK-, GFP-, or IBAR-GFP-transfected cells (Figure 2B), and then treated untransfected cells with the purified EVs. We detected significantly more cells expressing green fluorescence in the untransfected cells with EVs generated from IBAR-GFP-transfected cells than those from MOCK control or GFP alone-transfected cells (Figure 2C), strongly supporting that the IBAR domain of IRSp53 indeed induces EV production.

### IBAR-F3-GFP can induce the production of EVs loaded with activated integrin

**Figure 3.**
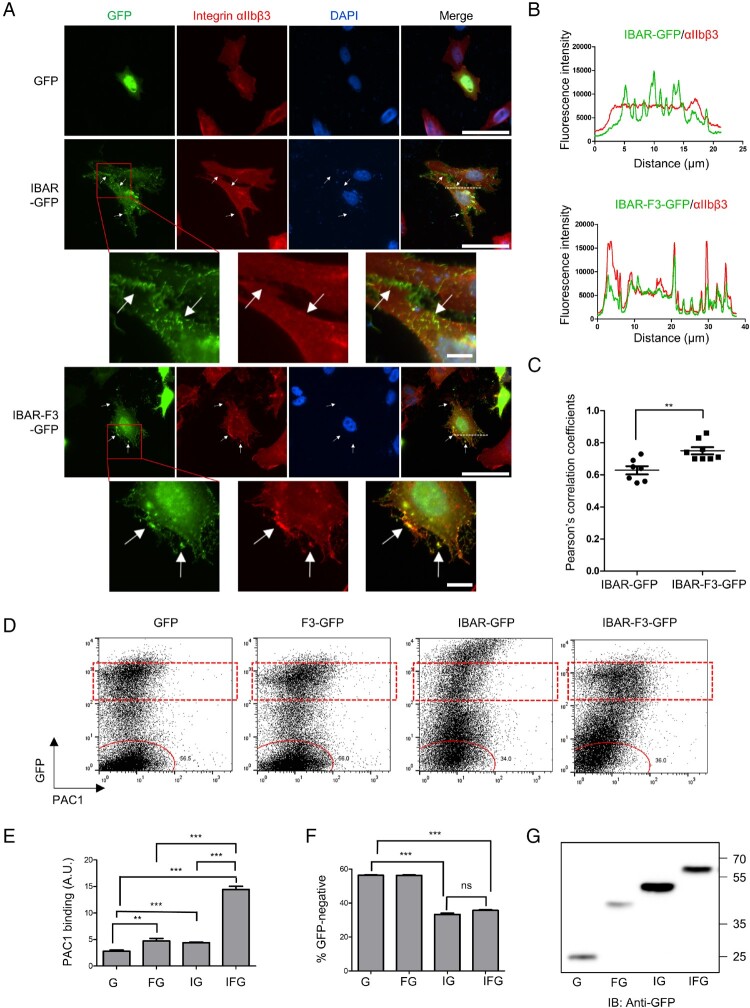
**IBAR-F3-dependent intercellular communication**. (A) CHO/αIIbβ3 cells transfected with GFP, IBAR-GFP, or IBAR-F3-GFP were stained with integrin αIIbβ3 antibody (D57) and Hoechst 33342. Stained cells were visualized by fluorescence microscopy, and representative images are shown. Scale bar, 50 µm. Digitally magnified images of the regions indicated with red dotted boxes are shown. Scale bar, 10 µm. White arrows indicate filopodia-like membrane protrusions. (B) The graph illustrates the fluorescence intensities of IBAR-GFP or IBAR-F3-GFP in comparison to those of integrin αIIbβ3 at the region indicated by the white dotted line in panel A. (C) Scatter plots illustrating Pearson’s correlation coefficients between the fluorescence intensities of the IBAR domain and integrin αIIbβ3 within individual cells were presented. Error bars represent SD (*n* = 7 and 8 cells for IBAR-GFP and IBAR-F3-GFP, respectively). **p < 0.01 (Student’s t-test). (D) CHO/αIIbβ3 cells transfected with GFP, F3-GFP, IBAR-GFP, or IBAR-F3-GFP were analyzed for the degree of integrin αIIbβ3 activation using an activated integrin-specific antibody, PAC1. Representative dot plots showing GFP fluorescence and PAC1 binding to cells in each condition are shown. The region of cells with moderate GFP expression levels is indicated with the dotted square, the untransfected populations are indicated with dotted circles. (E) The mean fluorescence intensity of PAC1 binding in the region indicated with the dotted square is shown in the bar graph (*n* = 3). Error bars indicate SD (*n *= 3). ***p < 0.001 (Student’s t-test). (F) The percentage of the GFP-negative population (cells in red circles in panel B) among total cells was calculated and shown as a bar graph. Error bars indicate SD. ***p < 0.001 (Student’s t-test). (G) CHO/αIIbβ3 cells transfected with various constructs were analyzed by western blot using anti-GFP antibody. G, GFP; FG, F3-GFP; IG, IBAR-GFP; IFG, IBAR-F3-GFP; GFP, green fluorescence protein.

Next, we asked whether the negative curvature induced by IBAR can facilitate activation of nearby integrins. Therefore, we proceeded to fuse the IBAR domain with the integrin β tail-binding talin F3 domain to ensure curvature of the membrane adjacent to the integrin. Similar to IBAR-F3, the introduction of IBAR-F3-GFP into CHO/αIIbβ3 cells induced extensive membrane protrusions without noticeable differences (Figure 3A). Additionally, IBAR-F3-GFP could recruit integrin αIIbβ3 near the IBAR-F3-GFP-induced membrane protrusion sites (Figure 3A) evident by fluorescence intensity line profiles (Figure 3B) and Pearson’s correlation coefficients (Figure 3C), demonstrating the interaction of IBAR-F3-GFP with integrin αIIbβ3. Importantly, upon analyzing IBAR-F3-GFP-transfected cells for their binding to an activation-specific integrin αIIbβ3 antibody, PAC1, we observed a substantial increase in PAC1 binding (Figure 3D and E), compared to IBAR-GFP- or F3-GFP-transfected cells. These data indicate that the negative curvature induced by the IBAR domain, which fused to F3, should facilitate integrin activation. Nonetheless, we do not exclude the possibility that F3 domain somehow become more potent in integrin activation when fused to IBAR, independent of the curvature effect. Interestingly, in the IBAR-F3-GFP transfection condition, the region corresponding to the untransfected population also shifted toward higher GFP expression (Figure 3D), resulting in a reduced percentage of the GFP-negative cell population, similar to that observed in the IBAR-GFP transfection condition (Figure 3F). Taken together, these results suggest that IBAR-F3-GFP can be utilized to produce EVs loaded with integrins which the IBAR-induced negative curvature can further activate. We also noted that the expression level of IBAR-GFP was consistently higher than that of IBAR-F3-GFP in our routine experiments, as analyzed by flow cytometry (Figure 3D) and western blot (Figure 3G). Nevertheless, the shift toward higher GFP expression was at similar levels in both cases (Figure 3F), implying that the EVs produced in the cells transfected with IBAR-F3-GFP may be more effectively uptaken by recipient cells than IBAR-GFP and that integrin loaded into the EVs by IBAR-F3 may contribute to the efficient uptake.

### Uptake of IBAR-F3-GFP-induced EVs depends on integrin

**Figure 4.**
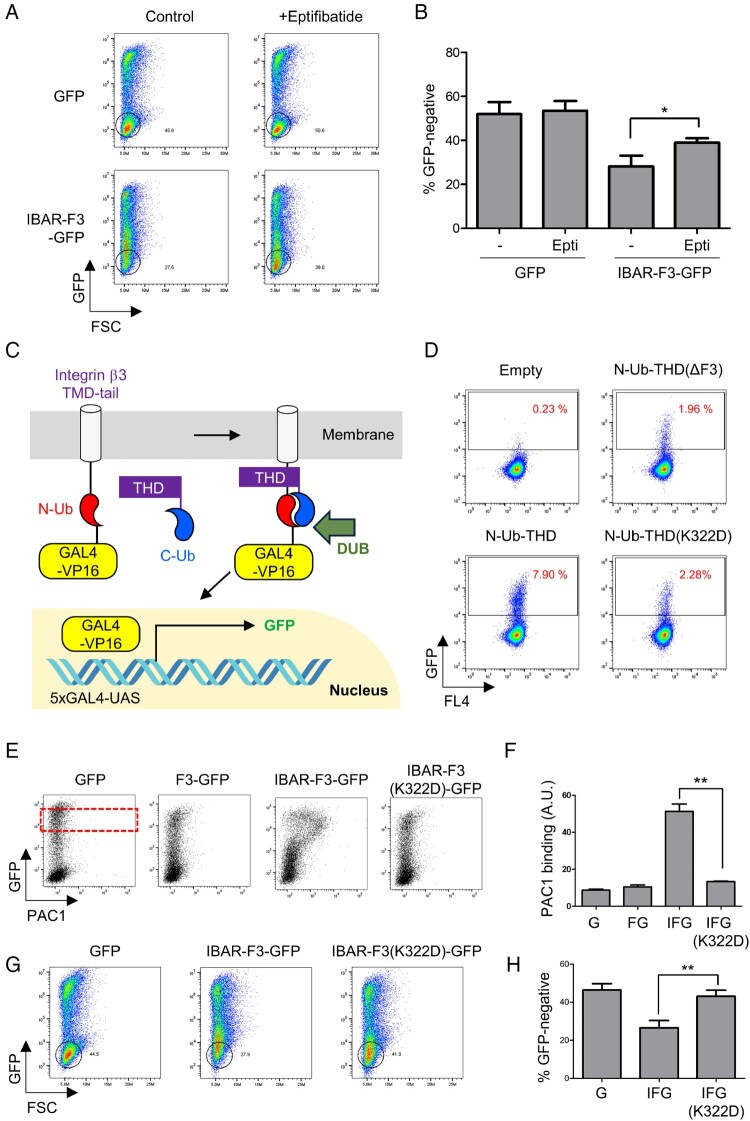
**Integrin-dependent uptake of IBAR-F3-GFP-induced EVs**. (A) CHO/αIIbβ3 cells transfected with GFP or IBAR-F3-GFP were treated with an integrin αIIbβ3-specific antagonist, eptifibatide (20 μM), and incubated overnight. The resulting cells were analyzed by flow cytometry. Representative dot plots are shown, as in Figure 1B. (B) The percentage of the GFP-negative population in (A) is displayed, as in Figure 1D. Error bars indicate SD. *p < 0.05 (Student’s t-test). (C) A schematic diagram of the mammalian two-hybrid assay is shown. In the assay, reassembly of ubiquitin by the interaction between integrin β3 and the talin head domain (THD) causes a deubiquitinating enzyme (DUB)-dependent liberation of GAL4-VP16, resulting in GFP expression. N-Ub, N-terminal half of ubiquitin; C-Ub, C-terminal half of ubiquitin. (D) CHO cells stably incorporated with 5xGAL4-UAS and the integrin β3 TMD-tail construct were transfected with N-Ub fused to either wild type, F3 deletion mutant (THDΔF3), or K322D mutant THD. The effects of transfection on GFP expression were analyzed by flow cytometry. The percentages of GFP-expressing cells in each condition are shown in the dot plots. (E, F) CHO/αIIbβ3 cells transfected with GFP, F3-GFP, IBAR-F3-GFP, or IBAR-F3(K322D)-GFP were analyzed for integrin αIIbβ3 activation, as in Figure 3D and 3E. Error bars indicate SD (*n* = 3). **p < 0.01 (Student’s t-test). (G, H) The percentage of the GFP-negative population in CHO/αIIbβ3 cells transfected with GFP, IBAR-F3-GFP, or IBAR-F3(K322D)-GFP was analyzed, as in Figure 1B and D. Error bars indicate SD (*n* = 3). **p < 0.01 (Student’s t-test). Epti, eptifibatide; FSC, forward scatter; GFP, green fluorescence protein.

Next, we tested whether EV uptake is dependent on integrin function. For this, CHO/αIIbβ3 cells transfected with either GFP or IBAR-F3-GFP were incubated for an additional 24 h in the presence or absence of 20 μM eptifibatide, a specific antagonist to integrin αIIbβ3. The treatment with eptifibatide suppressed the IBAR-F3-GFP-induced shift (Figure 4A), which was also confirmed by the significant increase in the percentage of GFP-negative cells (Figure 4B). Inhibitor treatment after transfection is generally not expected to block the process completely. To directly address the role of integrin in EV uptake, we used a mutant F3, which harbors the K322D mutation that blocks binding to integrin. In our integrin-talin binding assay, which was developed based on the mammalian two-hybrid assay (Petschnigg et al. 2014), the interaction between the C-terminal half ubiquitin-fused integrin β3 transmembrane domain-tail and the N-terminal half ubiquitin-fused THD triggers the reassembly of ubiquitin, which leads to the liberation of GAL4-VP16 by a deubiquitinating enzyme and initiated GFP expression (Figure 4C). Therefore, GFP expression serves as a readout for the interaction between integrin and talin. The percentage of GFP-positive cells was 7.90%, in case an intact wild type THD was used in the assay. However, it was only 1.96%, in case of the deletion mutant, THD(ΔF3), which lacks the integrin-binding domain F3 (Figure 4D). When the K322D F3 mutant was used, the percentage of GFP-positive cells was similar to that of THD(ΔF3) (Figure 4D). When the integrin-binding defective mutation, K322D, was introduced to IBAR-F3-GFP to generate the IBAR-F3(K322D) mutant, the activation effect was largely inhibited (Figure 4E, abscissa, and 4F), although the expression level of IBAR-F3(K322D) was similar to that of the wild type (Figure 4E, ordinate). In addition, the IBAR-F3-mediated shift to high GFP expression was also largely suppressed (Figure 4G and H), indicating the critical role of F3-mediated integrin activation on the efficient EV uptake. Altogether, these results suggest that expressing IBAR-F3 can produce EVs, which are efficiently transferred to recipient cells.

## Discussion

Our results suggest that the IBAR domain fused to the integrin-binding talin F3 domain, IBAR-F3, is useful for producing EVs, which can be efficiently uptaken by recipient cells. The IBAR domain in the fusion protein is likely to facilitate EV formation by inducing membrane protruding, while F3 domain linked to the IBAR recruits and activates integrins in the EVs.

The IBAR-F3 fusion protein in this study provides several advantages in terms of the usage of EVs. First, the IBAR-F3-mediated EV production can be utilized in various cell types expressing different integrins, because the talin F3 domain effectively binds to the β tail of most types of integrins (Calderwood et al. 2003; Lefort et al. 2012; Kukkurainen et al. 2014; Baade et al. 2019). Second, IBAR-F3 fusion increases EV uptake into recipient cells, dependent on integrin activation. The mechanism of integrin activation may occur through the IBAR-mediated negative membrane curvature formation near integrins, which may disrupt integrin α–β transmembrane domain interaction, leading to integrin activation by changing integrin β topology within the curved membrane, as observed in positive membrane curvature formation (Kim et al. 2020). Given the importance of integrin activation for ligand binding, the activated integrins on EVs induced by IBAR-F3 should greatly enhance EV uptake by binding to their ligands, extracellular matrix proteins, on the surface of recipient cells. Third, the IBAR-F3 domain can be further engineered by genetic fusion to contain other forms of protein cargo, which can then be delivered to the target cells.

Integrin-mediated cell adhesion is tightly regulated by signaling pathways in cells (Kim et al. 2011b; Ye et al. 2012). Integrin-mediated adhesion is crucial for directing EVs to specific recipient cells, as shown in numerous studies, thus it is likely that the machinery for integrin activation plays a critical role in the process. The incorporation of large-scale integrin signaling and activation machinery into nanoscale EVs might be significantly limited, especially in small EVs, particularly less than 50 nm in diameter. Interestingly, IBAR domains are known to induce a curvature with several tens of nanometers in radius (Nepal et al. 2021), which corresponds to the size of small-sized EVs. However, overexpression of the IBAR domain highly facilitates integrin activation, as shown in this study (Figure 3D), and integrins incorporated into small-sized EVs are presumably at a high-affinity state owing to the intrinsic membrane curvature, probably active even with no contribution from integrin signaling and activating machinery. Therefore, it is likely that integrin-containing EVs are still competent carriers without sacrificing cargo capacity.

In conclusion, this study presents an effective engineering procedure for producing EVs, which is highly efficiently transferred to recipient cells. Furthermore, our findings explain how integrins on EVs could be activated by a membrane curvature change. As a result, we propose that expressing IBAR-F3 in relevant donor cells could significantly enhance the production of EVs with high efficiency in cell-to-cell transfer customized for medical applications.
